# Septic arthritis in the pediatric hip joint: a systematic review of diagnosis, management, and outcomes

**DOI:** 10.3389/fped.2023.1311862

**Published:** 2023-12-21

**Authors:** Alessandra Nannini, Riccardo Giorgino, Luca Bianco Prevot, Andrea Bobba, Domenico Curci, Riccardo Cecchinato, Giuseppe M. Peretti, Fabio Verdoni, Laura Mangiavini

**Affiliations:** ^1^ Residency Program in Orthopedics and Traumatology, University of Milan, Milan, Italy; ^2^ IRCCS Ospedale Galeazzi Sant'Ambrogio, Milan, Italy; ^3^ Dipartimento di Scienze Biomediche per la Salute, Università Degli Studi di Milano, Milan, Italy

**Keywords:** septic arthritis, hip infections, pediatric hip arthritis, hip septic arthritis, orthopedic infections

## Abstract

Septic arthritis of the pediatric hip joint (SAH) is a rare but serious orthopedic emergency requiring immediate diagnosis and management. Delayed recognition can lead to severe complications, emphasizing the need for timely intervention. This systematic review aims to provide a comprehensive analysis of SAH in the pediatric population, focusing on its diagnosis, management, and outcomes. The review included 11 studies involving 391 patients with SAH, aged between three months and 12 years. Staphylococcus aureus was identified as the most common causative pathogen, with increasing cases of methicillin-resistant strains. Diagnosis is challenging due to nonspecific clinical presentations, necessitating validated criteria and a multidisciplinary approach. Ultrasound emerged as a valuable tool for early detection, and MRI was used in challenging cases. Treatment options include hip aspiration, arthrotomy, and arthroscopy, often combined with appropriate antibiotic therapy. Success rates were comparable among different surgical procedures. Early intervention is vital for optimal outcomes. However, the review highlights the need for standardized protocols and further prospective studies to address limitations and improve understanding and management of SAH in the pediatric hip joint.

## 1. Introduction

Septic arthritis of the pediatric hip joint (SAH) is an uncommon but severe condition considered an orthopedic emergency and requires prompt diagnosis and management. Delayed recognition and treatment of SAH can result in severe complications, including avascular necrosis (AVN) of the femoral head, osteomyelitis, chondrolysis, hip dislocation, leg-length discrepancy, systemic sepsis, and future osteoarthritic degeneration (1). Given the potential for devastating consequences, a comprehensive understanding of the diagnosis, management, and outcomes of SAH in the pediatric population is essential. SAH in the pediatric population is relatively rare, with an incidence reported to be approximately 1–10 cases per 100,000 individuals (2, 3). The hip is the most affected joint in septic arthritis (4). SAH can arise from various etiological factors, with bacteria being the most common causative agent. Staphylococcus aureus is the most common, with a recent increased incidence of the methicillin-resistant strain (5). The remaining cultured organisms are mainly Kingella kingae and Streptocococcus species (6). Also, fungi and viruses are among the causative pathogens of SAH (7).

Intra-articular infection can result from hematogenous spread, extension from a local infection (osteomyelitis), or direct inoculation of the joint. Direct inoculation may result from an open trauma but is rare (8). Hematogenous seeding of the joint seems to be the most frequent cause of SAH in the pediatric population (9) although precise prevalence data are not available in the literature. Interestingly hematogenous spread appears to be the most common cause worldwide, without distinction between developed and less developed countries (10). In Western and sub-Saharan African children SAH may be caused by a wide spectrum of bacteria, mainly group B Streptococci, followed by Staphylococcus aureus and gram-negative rods. Some authors also document a rise in joint infections caused by Kingella kingae, corresponding to the decrease in H. influenzae infections (11, 12).

Factors contributing to the onset of septic arthritis encompass youth (specifically, under the age of 3), male gender, prior trauma, and compromised immune function (13) such as in diabetes, HIV, drug and alcohol abuse. Regarding compromised immune status, numerous studies in the literature concentrate also on the association between sickle cell anemia and SAH (14–16).

The diagnostic challenge of SAH lies in its nonspecific clinical presentation, as hip pain and limping are relatively common findings in pediatric patients. Distinguishing between SAH and other hip conditions, such as transient synovitis, can be challenging, necessitating the use of validated diagnostic criteria and a multidisciplinary approach. Delayed diagnosis can result in significant morbidity, emphasizing the importance of accurate and timely identification of SAH (17).

Specific diagnostic criteria have been validated in the past (18) and recent works have aimed to revalidate these algorithms to understand whether they still play a role in diagnosis in the present days (19, 20). However, a prompt diagnosis can be challenging due to factors such as the absence of fever, normal leukocyte count, and negative C-Reactive Protein (CRP) or Erythrocyte Sedimentation Rate (ESR), which cannot be used to rule out septic arthritis (7). Moreover, the possible clinical presentations may vary according to age, type of infection, and etiology, so clinical suspicion must remain high in a child with hip pain and inability to walk (21). The diagnostic workup and definitive treatment require a multidisciplinary approach. Several algorithms have tried to standardize the diagnostic procedures and treatment of septic arthritis (22). However, a consensus has yet to be reached, probably due to the small number of patients included in the studies available. The most reliable method to diagnose SAH is through joint aspiration (23). A detailed history and physical examination should be performed on all pediatric patients with sudden hip pain. Doctors should pay particular attention to fever and the inability to bear weight. Complete blood count (CBC), ESR, and CRP level testing should be requested for any patient showing clinical symptoms suggestive of SAH (24). The Kocher criteria for SAH can be used for every child with an acutely inflamed hip (25). Sometimes, it can be difficult to differentiate between transient synovitis of the hip and septic arthritis, and these criteria can help to select subsets of patients who need urgent orthopedic attention (26). The four Kocher criteria are fever higher than 38.5 C, ESR more than 40, inability to bear weight, and white blood cell (WBC) count more than 12,000. Children who meet one out of four criteria have a 3% incidence of septic arthritis, 40% incidence with two out of four, 93% with three out of four, and 99% incidence with all four criteria (27). Radiographs are routinely obtained for patients experiencing hip pain and unable to bear weight, however they are marginal in evaluating and diagnosing patients with possible SAH (4). Radiographic characteristics of SAH encompass narrowing of the joint space, bilateral destruction of articular surfaces, and sclerosis. However, early diagnosis and treatment can prevent these changes to the joint (28). Ultrasound is now widely used in clinical practice since it is a safe and easy method to detect an effusion (29). More recently second-level imaging, in particular MRI, is getting more attention as a valuable tool to better detect the involved joint and any associated area of infection (17). Early and appropriate antibiotic therapy targeting the causative pathogen is crucial to eradicate the infection and prevent complications. Guidelines regarding the duration of intravenous (IV) antibiotic therapy differ according to the pathogen and the age of the patient (30). The most feared complication of SAH is avascular necrosis of the femoral head with an incidence reported in the literature between 20% and 28% (31, 32).

Surgical intervention, such as joint aspiration and drainage, is often necessary to confirm the diagnosis, relieve joint effusion, and minimize joint destruction (33). Surgical techniques, including arthrotomy and arthroscopy, are employed based on the severity and characteristics of the infection. Arthrotomic treatment remains the gold standard, but arthroscopy is increasingly used with good results (34).

While numerous studies have investigated SAH in the pediatric hip joint, there is a need to synthesize the existing evidence and provide a comprehensive analysis of the diagnosis, management, and outcomes of this condition. Therefore, the aim of this systematic review is to provide a comprehensive analysis of the diagnosis, management, and outcomes of septic arthritis in the pediatric hip joint and help guide clinicians in making evidence-based decisions regarding diagnosis and treatment.

## 2. Materials and methods

For this systematic review, we searched the electronic databases PubMed, Scopus, and Embase using the search terms: “hip” AND (“septic arthritis” OR “coxitis”) AND (“children” OR “newborn” OR “young adults” OR “infant”). The search was limited to articles published in the last 15 years to have updated results. Articles were reviewed according to the Preferred Reported Items for Systematic Reviews and Meta-Analyses Statement for Individual Patient Data (PRISMA-IPD). The research questions, inclusion, and exclusion criteria were decided *a priori*. The inclusion criteria included human studies published in English and studies focusing on risk factors, diagnosis, and management of septic arthritis of the native hip in the pediatric population. Exclusion criteria were septic arthritis in adults; avascular necrosis of the femoral head; rheumatic disease; septic arthritis involving other joints (knee, shoulder, sternoclavicular, wrist, pubic symphysis); tumors; hip dysplasia; studies on animals/cadavers; aseptic disease. After excluding the duplicates, two reviewers (AN, LBP) screened each study's title, abstract, and full text. Disagreements were solved by discussion after full-text evaluation. The reference lists of the studies were manually searched for other publications that may have eluded the initial search. Two reviewers (AN, LBP) independently evaluated each study for quality. The review protocol was registered with Prospero (CRD42023424760).

Statistical analyses were performed using GraphPad Prism, version 8.2.1 (GraphPad Software, Inc., La Jolla, CA, USA).

### 2.1. Quality assestment

Methodological quality of included studies was assessed independently by two separate authors (A.N and R.G.). The risk of bias was analyzed for each study, with the Methodological Index for Non-randomiyed Studies (MINORS) criteria (35). The MINORS tool is a validated instrument designed to assess the methodological quality of nonrandomized studies. The maximum score for non-comparative studies is 24.

## 3. Results

### 3.1. Study selection

**Figure 1:**
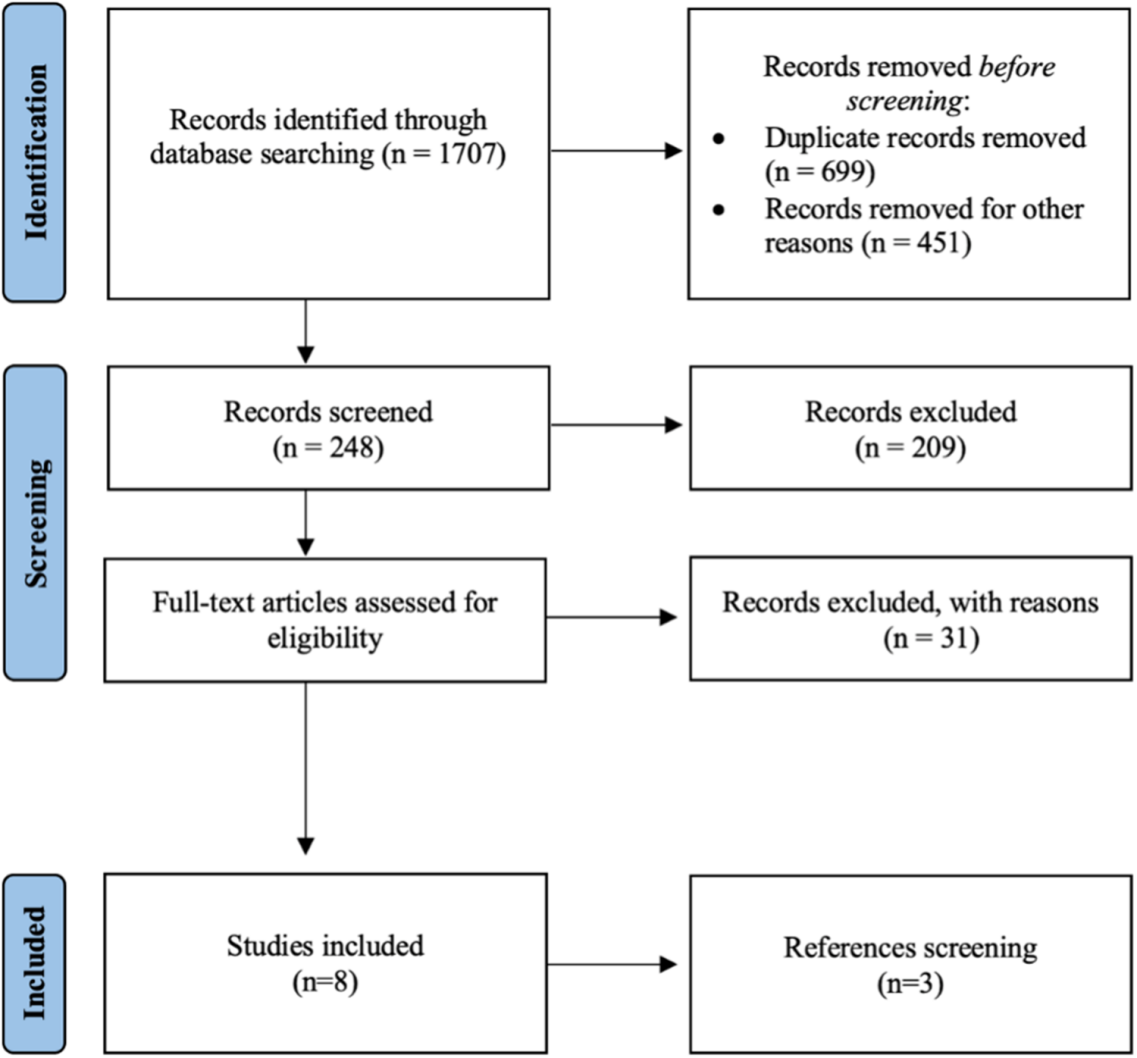
A flowchart of the literature screening performed in this study.

A total of 1,707 articles were retrieved for preliminary evaluation (Figure 1). After the duplicates were eliminated, 1,008 original articles remained. Two authors (AN, LBP) reviewed the titles: 1,459 studies were excluded because they did not meet the inclusion criteria or were published in a language other than English. After the abstracts were reviewed, 209 studies were excluded getting the number down to 39. Of the remaining articles, 31 were excluded after evaluating the full text. Three additional studies were found by manually searching the reference lists of the selected articles for an end total of 11 studies. The final analysis included retrospective case-control studies (*n* = 6), retrospective case series (*n* = 3), and a prospective study (*n* = 2). Due to their design only two studies have a level of evidence equal to 1, while the others have a level of evidence of 3 or 4. The inclusion of only two prospective study was the main study quality deficiency.

### 3.2. Risk of bias and study quality assestment

The risk of bias assestment for the studies was performed using MINORS criteria (35). MINORS scores range from 14 to 22, with an average score of 15.63. The major deficy was the Lack of a control group. All the studies showed a clearly stated aim, appropriate endpoints, and a small loss of follow-up. MINORS scores for the included studies are shown in Supplementary Table S1.

### 3.3. Study characteristics

All the studies reported data on diagnosing and managing septic arthritis in the pediatric native hip. A total of 391 patients were included, with a mean patient age of 5.7 ± 2.1 years (range 3 months–16.4 years), in the studies of Thompson et al. and Hoswell et al. age range was not available or inferable from the text. Female/male ratio was reported in nine out of 11 studies (300 patients) with a prevalence of male sex (54.3%). The right side seems to be slightly more affected than the left side (54%–66%) (4, 36, 37). The mean postoperative follow-up was 3.9 ± 2.8 years (range 1–8.5 years) (Table 1).

**Table 1:** Characteristics of the selected studies.

Authors	Country	Study type	Year	N° patients	Sex	Mean age (day, month, year)	Baseline mean PCR (Mg/Dl)	Most common pathogen (%)	Mean duration Of symptoms before treatment (day)	Treatment	Follow up (year)
Duman et al. (4)	Turkey	Re	2019	15	9 M 6 F	5.2 (2–10) year	58 (24–126)	S. Aureus	<5	A	2
Thompson et al. (8)	USA	Re	2015	9	5 M 4 F	5 year	/	S. Aureus + S. epidermidis	/	A	1.3
Sanpera et al. (34)	Spain	Re	2015	12	/	6.18 year (19 month−12 year)	13.35 (28.8–2.8)	S. Aureus (66.6%), S. Pyogenes (16.6%), Pneumococcus (8.3%), no diagnosis (8.5)	3.5	A	5
Pääkkönen et al. (37)	Finland	Pro Ran	2010	62	32 M 30 F	7.2 (4.4–11) year	9.1	S. Aureus 71%,, H. Influenzae Type B 10%, S. Pyogenes 10%, S. Pneumoniae 8%	3	Al, T	1 (Min)
El-Sayed et al. (25)	Saudi Arabia	Pro Ran	2008	20	11 M 9 F	8.05 (3–12) year	/	S. Aureus (55%)	2.9	A, T	2.26
Cohen et al. (33)	Israel	Re	2023	79	/	8 (2–14) year	/	/	/	T, Al, Al + T	2 (Min)
Hoswell et al. (36)	Australia	Re	2019	37	M 18 F 19	6.1 year	4.14	S. Aureus 30.4%, S. Pyogenes 17.4%, S. Pneumoniae 13%, K. Kingae 8.7%	4	T, Al	8.5
Lee et al. (38)	South Korea	Re	2015	31	M 17 F 14	3 month (10 day−18 month)	/	S. Aureus	4.32	T	6.2
Weigl et al. (39)	Israel	Re	2016	42	M 18 F 24	5.9 year (6 month−16.4 year)	8.2 (7–12.6)	Coagulase Positive S. Aureus	2.7	Al, Al + T	7.44
Journeau et al. (40)	France	Re	2011	43	M 29 F 11	5.3 year (3 day−14 year)	59.56 (2.5–336)	S. Aureus	4.09	Al, Al + T	/
Danilov et al. (41)	Germany	Re	2022	41	M 24 F 17	6.04 year (7 month−14 year)	8.12 (0.3–32)	S. Aureus	6.2	A, A + T	/

Re, retrospective study; Pro Ran, prospective randomized; M, males; F, females; y, years; m, months; d, days. A, arthroscopy; T, arthrotomy; Al, arthroscopic lavage.

### 3.4. Diagnosis and management of septic arthritis in the pediatric hip joint

The predominant causative organism for septic arthritis of the hip in the pediatric population is Staphylococcus aureus in its methicillin-sensitive variant (MSSA) ranging from 30% to 71% (4, 8, 25, 33, 34, 36–41). Streptococcus Pyogens is reported as the second most common causative pathogen with a prevalence between 17.4% and 10% (34, 36, 38), and Streptococcus Pneumoniae is reported in two studies with a prevalence ranging from 8.3% to 13% (34, 36). Only four studies included culture-negative infections with a range from 16% to 33% (8, 34, 38, 39). In the other studies, a negative culture was considered an exclusion criterion. The mean duration of symptoms at the time of surgery was 3.5 ± 0.9, ranging from 1 to 7 days. Diagnosis was based on one or a combination of clinical signs of infection.

Kocher's criteria to calculate the probability of septic arthritis were used in 3 studies (33, 37, 38) for a total of 161 patients. Caird's criteria were used in two studies (4, 40) for 57 patients and Newman's criteria were used in one study for 37 patients (36). Other criteria used are Morrey's Criteria in one study (31 patients) (39), and Blantyre septic joint score in one study (43 patients) (41). The rest of the patients were diagnosed with SAH based on a combination of lab tests, clinical symptoms, and imaging (8, 25, 34). CRP and ESR values at the time of diagnosis are reported just in 5 studies (4, 34, 37, 39, 40) and CRP alone in 2 studies (25, 36) with a mean CRP of 22.92 ± 24.64 mg/dl and mean ESR of 55.82 ± 16.3 mm/h (Table 2).

**Table 2:** Diagnostic criteria.

Authors	Criteria used to calculate the probability of SAH
Duman et al. (4)	Waldvogel criteria
Thompson et al. (8)	Preoperative serological markers, blood and/or joint aspirate cultures
Sanpera et al. (34)	Positive aspiration or positive blood culture without other sources of infection and/or leukocyte-rich aspirate
Pääkkönen et al. (37)	Kocher's criteria
El-Sayed et al. (25)	Kocher's criteria
Cohen et al. (33)	Kocher's criteria + diagnostic aspiration
Hoswell et al. (36)	Newman's criteria
Lee et al. (38)	Morrey Crit. + positive culture finding of joint fluid and WBCC 50.0 × 109 cells/L
Weigl et al. (39)	Caird crit. (Fever, CRP, ESR, WBCC, load inability)
Journeau et al. (40)	Blantyre septic joint score
Danilov et al. (41)	Load inability, limping or hip pain; elevated CRP, age over 4 years, symptoms for more than 4 days

SAH, septic arthritis of the hip.

Imaging was an integral part of diagnosis, and ultrasound was performed in all the included studies. A plain radiograph was performed in 9 out of 11 studies to look for soft tissue edema, synovial effusion, periosteal reaction, and bone degeneration; MRI was used just in the studies of Danilov et al. and Hoswell et al. to exclude adjacent osteomyelitis (36, 41). The main treatment targets in acute septic arthritis of the hip are sterilization and decompression. In 195 patients, hip aspiration and lavage was the main treatment (33, 36, 38, 40, 41). In 38 patients, a re-intervention was required: in those cases, the approach was arthrotomy (33, 38, 40, 41).

Hip arthroscopy was the treatment of choice in 88 patients (4, 8, 34, 41). A repetition was needed in three patients, and three underwent arthrotomy to improve their clinical condition (39–41). One hundred five patients were treated with hip joint arthrotomy as the first choice (33, 36, 37, 39). Antibiotic therapy was always associated with surgical procedures following different protocols according to the pathogen. The duration of antibiotic therapy ranged from 7 to 45 days. A study (4) reported the use of Cephazolin; a study used Clindamicine or a first generation Cephalosporine (37); a study used a first generation Cephalosporine (33); a study used Flucoxacilline (36); a study Cefuroxime (41); a study used selected therapy based on the Gram stain (34); another study reported the use of Cefotaxime and Fosfomicine if a child is under two years old or Methicillin and Gentamycin if older than three (40) (Table 3).

**Table 3:** Antibiotic therapy protocols.

Authors	Antibiotic	Duration of antibiotic therapy (day)
Duman et al. (4)	Cefazolin	34 (26–45)
Thompson et al. (8)	N/A	N/A
Sanpera et al. (34)	N/A	37.5 (30–45)
Pääkkönen et al. (37)	Clindamicin Or I Gen. Cefalosporin	20 (10–30)
El-Sayed et al. (25)	N/A	10 EV + 30 PO
Cohen et al. (33)	I Gen. Cefalosporin	3–16 EV + at least 2 PO
Hoswell et al. (36)	Flucoxacillin	30 (7 EV and 21 PO)
Lee et al. (38)	N/A	N/A
Weigl et al. (39)	N/A	21–42
Journeau et al. (40)	Cefotaxim + Fosfomicin If Or Meticillin + Gentamicin If 3/5 years old	N/A
Danilov et al. (41)	Cefuroxim	

d, days; N/A, not available.

Four papers reported the utilization of intraarticular drainage after the main procedure (8, 25, 38, 41). A total of 45 complications were reported (Table 4). Hospital stay changed according to the type of treatment with a mean of 7.7 ± 3.7 ranging from 3 to 46 days.

**Table 4:** Reported complications.

Complications	N. (tot. 45)
Avascular necrosis	1 (8)
Osteomyelitis	10 (37)
Femoral palsy	1 (8)
Radiological abnormalities	27 (36, 38)
Clinical disatisfaction	5 (38)
Acetabular lesion with clinical impingement	1 (40)

## 4. Discussion

This review aims to provide a comprehensive overview of the causes and development of septic arthritis in the hip joint among children, while also examining the existing diagnostic methods and available treatment options. Septic arthritis of the hip is a critical condition that necessitates immediate attention due to the significant impact that timely diagnosis and treatment can have on the patient's prognosis. Given the infrequent occurrence of this condition in the antibiotic era, the identification and diagnosis of SAH pose challenges. This difficulty can be amplified by the absence of distinct clinical indicators, particularly in younger patients who may be less cooperative. Regrettably, there is no single definitive test considered the gold standard for diagnosing SAH in children. Diagnosis relies on clinical signs of infection, which can be assessed individually or in combination (42). Typically, bacterial hip arthritis manifests with fever, intense pain, swelling, and restricted joint function, as reported in all reviewed studies. Assessing the child's weight-bearing status is paramount, as a limping child with hip pain during the clinical examination should always prompt further testing (41).

The studies in our review describe clinical, biological, and radiographic criteria to evaluate a child presenting to the Emergency Room (ER) with suspicion of SAH. Kocher's criteria remain the most valuable tool for distinguishing septic arthritis from transient synovitis (18). These criteria include non-weight bearing, fever >38°C, ESR >40 mm/h, WBC >12,000 cells/mm^3^. The modified version of Kocher's criteria, known as Caird's criteria, adds CRP >2 mg/dl as a fifth criterion (43). When all five parameters are present, the predictive accuracy for septic arthritis compared to transient synovitis exceeds 97.5% (4). These findings align with the literature, where elevated serum CRP and ESR are often included in clinical prediction algorithms (44–46). Moreover, multiple authors have demonstrated that CRP is a superior predictor of septic arthritis of the hip (43, 47).

Imaging continues to play a crucial role in the diagnosis of SAH, and various modalities are employed. While traditional radiographs are becoming marginal in evaluating and diagnosing patients with possible SAH (28), hip ultrasound has gained popularity as a valuable procedure due to its affordability, noninvasiveness, and lack of radiation exposure.

The literature consistently underscores the superior sensitivity and specificity of hip ultrasound in detecting hip effusions compared to x-rays (48). Our review further substantiates these findings, suggesting that ultrasound emerges as the preferred investigative tool, given its utility for follow-up assessments as well (33). While Danilov et al. employed MRI scans in complex cases involving patients with weight-bearing issues, normal or elevated CRP levels, and prior ultrasound examinations, opting for MRI in young children can be intricate due to the need for sedation. However, it remains a pivotal resource for obtaining additional insights (36, 41). In a recent review and meta-analysis by Adam et al., MRI findings, particularly related to bone marrow changes, have played a crucial role in distinguishing septic arthritis from transient synovitis (49). Similarly, Kang et al. reported comparable outcomes, highlighting the importance of correlating these MRI findings with the patients' symptoms (50).

Our findings corroborate existing literature on the disease's etiology, highlighting S. Aureus as the primary causative agent, with Methicillin-sensitive Staphylococcus aureus (MSSA) being the most commonly identified strain. However, there has been a recent increase in the prevalence of methicillin-resistant Staphylococcus aureus (MRSA), which is a significant consideration impacting treatment strategies (8). Hematogenous transmission to the joint is the most frequent cause of SAH according to the literature (51). Among the studies included in our analysis, only one focused on the pathogenesis of the disease, confirming the prevalence of hematogenous spread (36). While some conditions such as underlying respiratory diseases and congenital anomalies have been identified as risk factors for septic arthritis of the hip in children, they seem to not influence the infection's outcome (36, 37, 39).

On the other side, a worse prognosis is associated to high values of CRP and ESR, delay in treatment (more than 6 days), and young age (34, 39, 41).

Hospital admission is always recommended for children with SAH. Orthopedic surgeons have various options available when dealing with it, the choice depends on the experience of the treating team and condition background (52). Based on the studies included in our review, three main approaches were recommended: arthroscopic debridement/lavage in single or repeated surgical times, arthrotomy, and needle aspiration-irrigation under general anesthesia. These procedures can be performed alone or in combination. These treatment modalities offer different benefits and considerations for the management of SAH in pediatric patients.

Hip joint arthrotomy remains the gold standard for the treatment of septic arthritis of the hip (SAH) (53, 54). It is the second most employed treatment in our review (25, 33, 36, 38). This procedure involves incising the joint capsule, draining the joint, thoroughly debriding the affected area, and irrigating it with an ample amount of normal saline solution (25). Nevertheless, there has been a recent shift towards less aggressive surgical interventions. While arthrotomy remains the standard of care, there is a growing interest in evaluating the potential side effects associated with soft tissue dissection compared to the minimally invasive serial aspiration-lavage technique (25). For revision surgery, arthrotomy is still the most common approach (33, 37, 39–41). In most patients, the initial treatment for SAH involves hip aspiration and lavage (33, 36, 37, 39, 40). This emergency procedure is performed in the operating room under general anesthesia, utilizing either the obturator internus or anterior approach based on the surgeon's preference, with needle aspiration guided by fluoroscopy or sonography. The attending surgeon chose the needle size; an 18-gauge needle (large enough for pus) was usually used, and the fluid obtained was sent for analysis (37, 39). If the fluid is purulent, the joint is irrigated with sterile saline until clear fluid is obtained (40). While arthroscopic debridement is a commonly accepted primary treatment for septic arthritis of the knee (SAK) in pediatric patients, its usage for SAH is not widespread. Open arthrotomy and debridement are considered the primary treatments for SAH (4). In cases where arthroscopic surgery is employed, the patient is placed under general anesthesia in a supine position, with the hip maintained at approximately 90 degrees of flexion and 40–60 degrees of abduction. Continuous traction is usually not required (4). A 5.5 mm arthroscope, along with two portals, an anterolateral portal and a lateral portal above the trochanter for irrigation, are typically used. The hip joint is thoroughly inspected, and the condition of the articular cartilage is assessed. Clear normal saline is used for irrigation until the return fluid becomes clear. Synovial samples may be collected for culture if necessary, and an aspiration drain is inserted (4, 8, 34, 41). In the reviewed studies, antibiotic therapy was combined with surgical procedures, with different protocols depending on the pathogen. The duration of antibiotic treatment is variable and different antibiotics were used based on gram stain results. Since a Gram-positive micro-organism is the causative pathogen in most children a β-lactam antibiotics can be commenced as first-line treatment in the absence of allergyn (55). Intraarticular drainage was utilized in four papers, with durations ranging from 1 to 3 days (8, 25, 41) or up to 2 weeks (38). Successful treatment, defined as the eradication of infection after antibiotic discontinuation, was reported in all the papers included in this systematic review. Among the reported complications, there was one case of avascular necrosis and one instance of spontaneously resolved femoral nerve palsy (8). Additionally, some patients experienced long-term functional limitations (36, 38).

The success rate is quite similar among the different surgical procedures, with needle aspiration-irrigation as the procedure with the highest failure rate. On the other hand, it is the less invasive procedure with the shortest hospital stay. All three treatment options are considered safe and well-established procedures within the medical community.

Remarkably, existing literature lacks conclusive evidence delineating the superior treatment modality for pediatric septic arthritis of the hip; a comprehensive systematic review conducted by Kang et al. in 2009 failed to yield definitive guidance on the preferred treatment approach for this condition with the authors suggesting a multidisciplinary approach involving surgeons, pediatricians, radiologists, microbiologists, and nurses (56). A more recent systematic review by Caldaci et al. emphasizes that there are no statistically significant differences regarding the outcomes and complications among various surgical techniques used in the included studies. In light of this, the choice appears to be purely operator-dependent (35).

Regardless of the specific treatment method chosen, the significance of early intervention is consistently emphasized as the most critical factor in attaining a favorable outcome for patients with SAH (8, 25, 34, 38).

This systematic review presents several limitations that need to be addressed. Firstly, the inclusion of only two prospective studies may limit the generalizability of the findings. Incorporating more diverse and representative studies would strength the overall validity and reliability of the conclusions. Additionally, a significant drawback of this review is the lack of standardization across the various papers examined. Differences in joint damage assessment, host characteristics, pathogen profiles, and the diverse techniques utilized for diagnosis and management introduce substantial heterogeneity among the included studies. This heterogeneity also complicates determining the optimal diagnostic approaches, treatment modalities, and long-term outcomes of septic arthritis in the pediatric hip joint. Another limitation is the potential for publication bias. Systematic reviews heavily rely on published studies, and there is a possibility that studies with negative or inconclusive results were not included, leading to an overestimation of treatment effectiveness or favorable outcomes. This bias may impact the overall conclusions and recommendations provided by the review.

Future research should address these limitations by incorporating a wider range of prospective studies with larger sample sizes. This would increase the diversity and representativeness of the data, leading to more robust and reliable conclusions. Additionally, implementing standardized protocols and criteria for assessing joint damage, host characteristics, pathogen profiles, and technique selection would enhance the comparability and reliability of results across different studies. By addressing these concerns, we can achieve more comprehensive findings that significantly contribute to the understanding and improvement of diagnosis, management, and outcomes of septic arthritis in the pediatric hip joint. Furthermore, conducting studies that address potential publication bias and ensuring the inclusion of studies with negative or inconclusive results would provide a more balanced perspective on the topic.

## 5. Conclusions

In conclusion, this study highlights key aspects of SAH. Swift diagnosis and treatment significantly impact outcomes. Staphylococcus aureus, especially methicillin-sensitive strains, prevails, but resistant strains are increasing. Early treatment is crucial; delays, high CRP/ESR levels, and younger age correlate with worse outcomes. Accurate diagnosis relies on clinical signs and ultrasound. Treatment options include surgery and less invasive methods, often combined with tailored antibiotics. Antibiotic resistance poses a challenge, requiring ongoing vigilance. Further research is warranted to address the evolving landscape of antibiotic resistance and explore potential interventions to improve outcomes in SAH patients.
