# Supplementary material for: Septic arthritis in the pediatric hip joint: a systematic review of diagnosis, management, and outcomes
Source: Front Pediatr. 2023 Dec 21;11:1311862. doi: 10.3389/fped.2023.1311862 (PMC10771295; doi:10.3389/fped.2023.1311862)
Supplement: Supplementary file 1 [file Table1.pdf]

Supplementary Table 1.

**Authors**	***Clearly stated aim***	***Inclusion of consecutive patients***	***Prospective collection of data***	***End points appropriate to aim of study***	***Unbiased assessment of the study end point***	***Follow up period appropriate to aim of study***	***Loss to follow up <5%***	***Prospective calculation of study size***	***An adequate control group***	***Contemporary group***	***Baseline equivalent of groups***	***Statistical Analysis***	***MINORS score***
Duman Et Al. [2]	2	0	0	2	2	2	2	0	N/A	2	N/A	2	14
Thompson Et Al. [6]	2	0	0	2	2	2	2	0	N/A	2	N/A	2	14
Sanpera Et Al. [18]	2	0	0	2	2	2	2	0	N/A	2	N/A	2	14
Pääkkönen Et Al. [20]	2	2	2	2	2	2	2	2	N/A	2	N/A	2	20
El-Sayed Et Al. [14]	2	2	2	2	2	2	2	0	2	2	2	2	22
Cohen Et Al. [17]	2	0	0	2	2	2	2	0	2	2	2	2	18
Hoswell Et Al. [19]	2	0	0	2	2	2	2	0	N/A	2	N/A	2	14
Lee Et Al. [21]	2	0	0	2	2	2	2	0	N/A	2	N/A	2	14
Weigl Et Al. [22]	2	0	0	2	2	2	2	0	N/A	2	N/A	2	14
Journeau et Al. [23]	2	0	0	2	2	2	2	0	N/A	2	N/A	2	14
Danilov Et Al. [24]	2	0	0	2	2	2	2	0	N/A	2	N/A	2	14
